# Inhibition of Crystal Growth during Plasma Enhanced Atomic Layer Deposition by Applying BIAS

**DOI:** 10.3390/ma8115425

**Published:** 2015-11-18

**Authors:** Stephan Ratzsch, Ernst-Bernhard Kley, Andreas Tünnermann, Adriana Szeghalmi

**Affiliations:** 1 Institut für Angewandte Physik, Friedrich-Schiller-Universität Jena, Max Wien Platz 1, Jena 07743, Germany; stephan.ratzsch@uni-jena.de (S.R.); ernst-bernhard.kley@uni-jena.de (E.-B.K.); Andreas.Tuennermann@iof.fraunhofer.de (A.T.); 2 Fraunhofer-Institut für Angewandte Optik und Feinmechanik, Albert-Einstein-Str. 7, Jena 07745, Germany

**Keywords:** plasma enhanced atomic layer deposition, titanium dioxide, anatase, plasma parameters, ion energy and nucleation, BIAS

## Abstract

In this study, the influence of direct current (DC) biasing on the growth of titanium dioxide (TiO_2_) layers and their nucleation behavior has been investigated. Titania films were prepared by plasma enhanced atomic layer deposition (PEALD) using Ti(OiPr)_4_ as metal organic precursor. Oxygen plasma, provided by remote inductively coupled plasma, was used as an oxygen source. The TiO_2_ films were deposited with and without DC biasing. A strong dependence of the applied voltage on the formation of crystallites in the TiO_2_ layer is shown. These crystallites form spherical hillocks on the surface which causes high surface roughness. By applying a higher voltage than the plasma potential no hillock appears on the surface. Based on these results, it seems likely, that ions are responsible for the nucleation and hillock growth. Hence, the hillock formation can be controlled by controlling the ion energy and ion flux. The growth per cycle remains unchanged, whereas the refractive index slightly decreases in the absence of energetic oxygen ions.

## 1. Introduction

Due to its remarkable properties, titania (TiO_2_) is a very interesting material for thin film applications. Its excellent transmittance in visible and infrared spectrum (VIS and IR), high refractive index, and chemical stability are attractive features for optical coatings [1,2]. Generally, titania has three different crystalline phases: brookite, anatase and rutile. Photocatalytic applications require anatase thin films or anatase nanoparticles with high surface-area-to-volume ratio [3,4,5]. A high surface-area-to-volume ratio of crystalline TiO_2_ results in a high surface roughness leading to high optical losses due to stray light. Thin films in optical applications require smooth surfaces. Therefore, the TiO_2_ amorphous phase is often preferred in optical devices, although crystalline TiO_2_ provides higher density and higher refractive index.

Various techniques to deposit thin TiO_2_ films have been applied e.g., chemical and physical vapor deposition (CVD and PVD) or atomic layer deposition (ALD) [6,7,8,9,10]. Especially, the interest of depositing thin TiO_2_ films with ALD has been growing over the past years [11,12,13,14]. Atomic layer deposition is a self-limiting, cycle-based coating technique, which enables to coat structured surfaces conformally with the desired material, e.g., for functional coatings on nanostructured surfaces [15,16,17,18,19,20]. Furthermore, this deposition technique allows excellent film uniformity and thickness scalability. Titanium dioxide can be deposited by thermal ALD as well as by plasma enhanced atomic layer deposition (PEALD) using a variety of metal halide and metal organic precursors. Crystalline TiO_2_ or crystalline grains embedded in amorphous layers are deposited at relatively low temperature in general above 150 °C [21,22]. Recently, we have reported on the formation of crystalline grains (=anatase hillocks) at 70 °C in PEALD process under specific oxygen plasma conditions [23].

Plasma enhanced atomic layer deposition provides the possibility to extend the atomic layer deposition temperature window to room temperature [24]. This feasibility opens a wide range of applications based on coating temperature sensitive materials with thin, high quality films. Room temperature deposition is enabled by highly reactive species generated in the plasma, such as radicals, ions, electrons, and photons. By controlling their flux and energy, film properties can be tailored for specific applications [23,25,26,27,28]. In remote inductively coupled plasma (remote-ICP), the plasma properties, such as ion energy distribution function (IEDF), electron temperature (*T*_e_), ion and electron density (*n*_i_ and *n*_e_, respectively) depend mainly on plasma gas pressure and composition, applied plasma power and the chamber configuration. Additionally, applying an external BIAS voltage to the substrate, the ion energy at the surface reaction site can be precisely controlled. In contrast to ALD, other deposition technologies like CVD or PVD commonly take advantage of controlling the ion energy and flux by applying a BIAS voltage [29,30,31].

The hillocks on the TiO_2_ surface considerably increase the surface roughness, which results in very high stray light losses [23]. Coinstantaneous they increase the effective surface area of the thin film which might be beneficial for photocatalytic applications. Accordingly it is the aim of this article, to evaluate which component in oxygen plasma, e.g., VUV-photons, electrons or ions, is responsible for their nucleation and the crystallite growth on TiO_2_ thin films. By controlling their flux and energy, it should be possible to control the nucleation and growth of crystallite for either photocatalytic or optical applications. First, the principles of plasmas and biasing are introduced. Then, the effect of direct current potential (DC) biasing on the TiO_2_ film morphology is shown in comparison to a TiO_2_ coating without DC-biasing. Based on these observations, the component in oxygen plasma is identified, that leads to the hillock formation in TiO_2_ during the plasma enhanced deposition. Finally, hillock free deposition is presented and characterized.

## 2. Results and Discussion

In the remote ICP deposition tool, the plasma source is mounted separately above the reaction chamber. It generates oxygen plasma, which is introduced to the chamber by a downstream flow. The oxygen plasma consists of radicals, ions, electrons, ozone, and oxygen, which can be either in excited or ground state [32]. It is assumed, that only three free charged carriers exist in the plasma, O_2_^+^ created by electron impact, O^−^ created by dissociative attachment of oxygen molecules, and electrons. In this plasma no thermal equilibrium occurs. The electrons have a higher temperature (*T*_e_ up to several eV) than the ions and neutrals components (*T*_i_ ≈ 25 meV), respectively. Since the electrons are at least three orders of magnitudes lighter, they have a significantly higher velocity than the ions. Consequently, they first escape from the plasma near the reactor walls. The plasma will be charged positively with respect to the reactor walls. As a result, a potential between the plasma and the walls, the self-BIAS potential emerges, which accelerates the ions towards the walls and deflect the electrons. The zone between plasma and walls is called plasma sheath. Because of the potential in the plasma sheath the electron and ion current is balanced. The maximum ion energy (*E*_i_) can be determined using the self-BIAS potential (*U*_self-BIAS_), which is the difference between the plasma potential (*U*_P_) and the substrate potential (*U*_S_): *E*_i_ = *eU*_self-BIAS_ = *e* (*U*_p_ − *U*_S_). Generally, the substrate potential is grounded (*U*_S_ = 0 V). Negative ions are trapped inside the plasma gas, due to their higher mass and lower energy compared to the electrons.

The properties of the oxygen plasma in a deposition tool, similar to the one used in this study was detailed by Profijt *et al.* [33]. They analyzed the kinetic energy and the flux of the ions with retarding field energy analyzer and tungsten planar current collecting probe, respectively. Further, they determined the electron temperature and electron density by double Langmuir probe measurement. Light emission of the oxygen plasma was also investigated. At higher chamber pressure, the ion energy and ion flux as well as the electron temperature and electron density tend to decrease. From their data no statements or extrapolation concerning the peak ion energy in the considered pressure range between 5.7 Pa and 24.0 Pa (corresponding to the chamber pressure of oxygen gas flow rate between 10 sccm and 100 sccm) can be made. Additionally, it was determined that high intensity vacuum ultraviolet light (VUV light) is emitted by the oxygen plasma. The authors pointed out that an VUV-exposure during the deposition can lead to defects or harm the coating [33].

**Figure 1:**
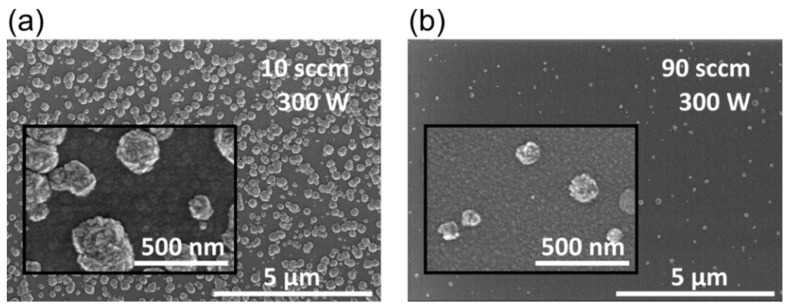
SEM images of the surface of TiO_2_ layers deposited on Si substrate under the following process conditions: 300 W plasma power; 100 °C substrate temperature; 10 sccm (**a**), and 90 sccm (**b**) oxygen gas flow corresponding to 5.7 Pa and 22.6 Pa chamber pressure, respectively.

Figure 1 depicts the surface of TiO_2_ layers that were deposited on Si substrates by two different oxygen gas flow rates (10 sccm (a); and 90 sccm (b)) during the oxygen plasma pulse. For both depositions, 5000 PEALD cycles were performed that lead to a thickness of (a) 170 nm; and (b) 160 nm, respectively. Numerous hillocks like anatase crystallites can be observed at 10 sccm, and a few are still growing at 90 sccm oxygen gas flow. These hillocks grow randomly on the TiO_2_ surface and their height strongly depends on chosen oxygen plasma conditions and deposited layer thickness. In a former study we classified these hillocks as the upper part of anatase crystallites surrounded by amorphous TiO_2_ layer [23]. We showed a correlation between the self-BIAS voltage for different plasma conditions and the formation of anatase hillocks on TiO_2_ layers at these plasma conditions [23]. Generally, high oxygen partial pressure in the plasma leads to low plasma potential which consequently causes lower maximum ion energy [32]. A high self-BIAS voltage results in numerous crystallites. However other particles, such as electrons or radicals, or photons might also activate the anatase phase growth during the deposition. Their energy or intensity might have a similar correlation to the flow rate. Here, a direct current potential is applied to the substrate, to reduce possible harmful effects of an ion bombardment.

**Figure 2:**
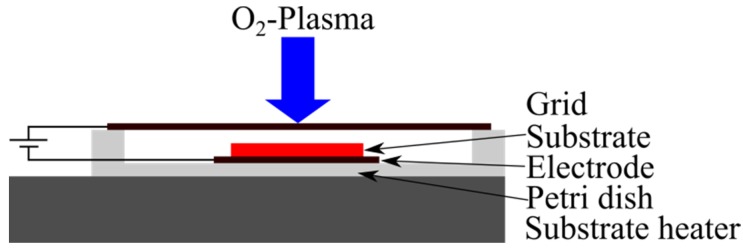
DC BIAS configuration in the reaction chamber.

The DCBIAS configuration is shown schematically in Figure 2. It was placed in the reaction chamber on the substrate heater. This configuration takes into consideration that a relatively high density of negative ions is formed in the oxygen plasma during the deposition [32]. Positively charged ions will be decelerated by applying a positive direct current potential (DC) at the electrode. If the substrate potential is sufficiently high (>plasma potential) the positive ions will be deflected. Simultaneously, electrons and negatively charged ions are attracted by the positive electric field and gain additional kinetic energy. To prevent the negatively charged ions from impinging on the substrate a metal grid is placed over the substrate. Electrons are able to escape from the plasma gas through the grid. As a consequence the plasma gas is charged positive *versus* the grid and so the negatively charged ions are trapped in the plasma gas. Electrons are accelerated towards the electrode and lead to a current flow. For that reason, it can be assumed, that no negatively charged ions reach the electrode or the substrate inside the DC BIAS configuration if a positive voltage is applied. Meanwhile radicals, ozone, oxygen molecules as well as VUV-light are unaffected by the electric field. They are available for further surface reactions.

**Figure 3:**
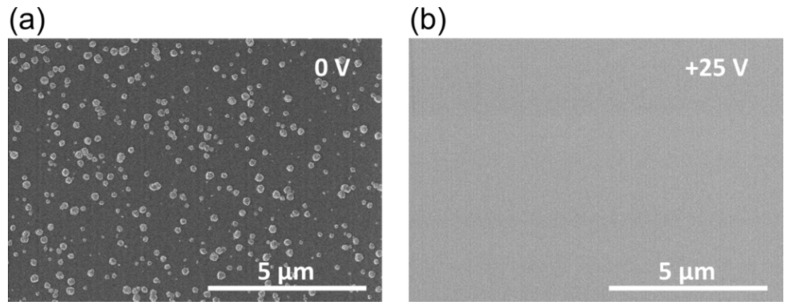
SEM images of TiO_2_ layers deposited on Si substrate under the following process conditions: 300 W plasma power; 100 °C substrate temperature; 10 sccm oxygen gas flow corresponding to 5.7 Pa chamber pressure and DC BIAS (**a**) 0 V; and (**b**) +25 V.

Figure 3 highlights the change in the TiO_2_ layer surface morphology deposited in the BIAS configuration at different applied voltage. In both cases 5000 PEALD cycles were performed on Si substrates. The oxygen gas flow amounts 10 sccm while the plasma power was 300 W during the oxygen plasma pulse. All other deposition parameters are identical to the deposition in Figure 1a, which will be labelled as reference in the following.

**Figure 4:**
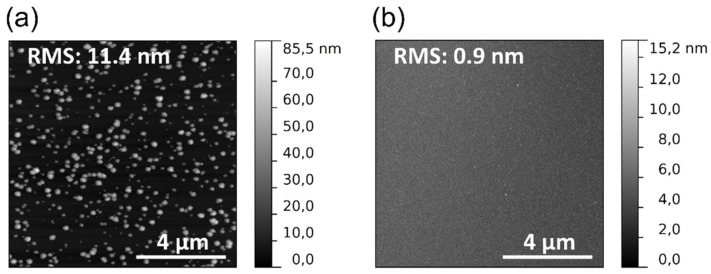
AFM measurement of the surface of TiO_2_ layers deposited on Si substrate under the following process conditions: 300 W plasma power; 100 °C substrate temperature; 10 sccm oxygen gas flow corresponding to 5.7 Pa chamber pressure at DC BIAS (**a**) 0 V; and (**b**) +25 V.

Compared to the reference surface in Figure 1a, the hillock density is reduced for both depositions within the DC BIAS configuration. As shown in Figure 1a many surface defects in the shape of hillocks have been formed on the reference surface. Their diameter and height is up to 300 nm and 120 nm, respectively. The hillock density and lateral size reduce for the sample shown in Figure 3a, mainly due the shielding and shadowing effect of the metal grid. Only a fraction of electrons, ions and photons reaches the surface, while the substrate is beneath the metal grid. Applying a positive voltage (+25 V) there are no hillock-like defects on the surface. The surface seems smooth which was verified by an atomic force microscope (AFM) measurement. The results of the AFM measurements are shown in Figure 4. No detectable hillock could be observed on the surface of the TiO_2_ layer that was deposited at DC BIAS +25 V. Consequently, the RMS of this TiO_2_ surface is much lower than the RMS of the TiO_2_ surface deposited at DC BIAS 0 V.

The layer properties are summarized in Table 1. Noticeable is the constant TiO_2_ layer film thickness regardless of the interference in the chamber geometry due to the introduction of DC BIAS configuration or applying a positive BIAS voltage during the deposition. Especially oxygen radicals combust isopropanol ligands during the plasma pulse [34]. They are uninfluenced by the DC BIAS, which causes a similar growth rate in the saturated regime of the ALD process. Also the surface reactions at the metal organic precursor pulse are unaffected by the DC BIAS.

**Table 1:** Film properties of the reference sample, and the layers deposited at DC BIAS 0 V and +25 V.

TiO_2_ Layer Properties	Reference	0 V	+25 V
Thickness (nm)	171 ± 2	167 ± 2	172 ± 2
Max height (nm)	120 ± 10	80 ± 8	15 ± 1
RMS (nm)	23.2	11.4	0.9
Hillock surface coverage (%)	52.8 ± 5.0	14.5 ± 1.5	–
Refractive index (λ = 1030 nm)	2.37 ± 0.02	2.37 ± 0.02	2.33 ± 0.02

The surface morphology of the films has been quantified by AFM measurements and by analysis of the scanning electron microscope (SEM) images in Figure 1 and Figure 3. The maximum height of the hillocks for samples deposited under reference conditions is (120 ± 10) nm. It decreases to 84 nm and 15 nm if the Si substrate is within the DC BIAS configuration at 0 V and +25 V voltages, respectively. The same trend is observed for the surface roughness (RMS) of the coatings. While the reference has an RMS of 23.2 nm, it reduces to 11.4 nm within the DC BIAS configuration and to 0.9 nm at DC biasing of +25 V. Likewise, the hillocks surface coverage decreases. The hillock surface coverage takes all hillocks which are shown in the SEM images into account and counts the ratio between hillock free and hillock covered surface. At the reference the coverage amounts over 50% while it decreases to 14.5% when titania is deposited within the DC BIAS configuration at 0 V. Obviously, no hillocks appear in the deposition at +25 V biasing.

The plasma potential for the applied oxygen plasma flow rate is (10.0 ± 0.5) V [23]. Hence, the applied DC BIAS of +25 V prevents the positively charged ions from reaching the substrate placed on the electrode. Electrons will gain extra kinetic energy. Consequently they impinge on the surface with a higher kinetic energy than without the applied DC voltage of +25 V. If they would harm the TiO_2_ layers, hillocks would also grow on the surface at the deposition by +25 V. Both the emission and the propagation of VUV light are not affected by the applied DC voltage. Accordingly, VUV light will reach also the surface even though with a lower intensity because of the shadowing effect of the metal grid. Based on these experiments, it seems likely, that ions are responsible for the nucleation and further hillock growth; whereas electrons and VUV light do not influence the hillock formation. It seems possible to control the nucleation behavior of TiO_2_, which is deposited by PEALD, by controlling the ion energy and ion flux during the plasma pulse. A high ion energy and ion flux lead to a high formation rate and growth rate of the anatase crystallites in the amorphous TiO_2_ film. They appear as hillocks on the surface and so they roughen it. This fact might be disadvantageous for an optical application of this thin film, but since anatase accrues, it could be beneficial in photocatalytic applications. The defect-free thin films could be used as optical thin film coatings. Therefore, the refractive index at 1030 nm was determined by Cody Lorentz oscillator model from the ellipsometer data. The absence of ions leads to a slight decrease in the refractive index at 1030 nm. From other deposition techniques it is well known, that energetic ion bombardment causes denser films with a higher refractive index [35].

## 3. Experimental Section

### 3.1. Deposition of TiO_2_-Layer

Titanium dioxide films have been deposited on p-doped Si(100) wafer. All substrates were thoroughly cleaned with isopropanol in ultrasonic water bath and deionized water. The residual organic solvents and water were removed by a strong stream of nitrogen prior to use. Titanium tetraisopropoxide (TTiP) was used as metal organic precursor and plasma activated oxygen as oxidizer. The duration of TTiP pulse was 1.5 s and the duration of oxygen plasma pulse was 8 s. The oxygen gas flow rate was 10 sccm at 300 W plasma power. The purge duration after the TTiP and O_2_-plasma pulse were 7 s and 4 s, respectively. In this study, 5000 cycles are deposited on each substrate. All depositions were performed at 100 °C substrate temperature, while the operating pressure alters between 5.3 Pa to 40.0 Pa.

The deposition was carried out in an open load ALD system from Oxford Instruments Plasma Technology, Yatton, UK. In the plasma enhanced deposition mode, the plasma is generated remotely in a separated chamber above the reaction chamber.

During the deposition within the DC BIAS configuration the sample was placed on the electrode. A Petri dish insulates the electrode from the grounded substrate heater. The metal grid is located 20 mm above the electrode on top of the Petri dish. Both, electrode and metal grid were connected to a direct current voltage generator placed outside the reactor chamber via electrical feedthrough, whereby the electrode is always connected to the anode. The metal mesh has a diamond-shaped pattern with a mesh size in the diagonals of 2 mm and 1.5 mm. It has no electrical connection to the grounded reaction chamber.

### 3.2. Characterization of TiO_2_-Layer

The film thicknesses were analyzed by spectroscopic ellipsometry (Woollam Inc., Lincoln, NE, USA M-2000 instrument, spectral range: from 246 nm to 1687 nm) and confirmed by measurements performed at SEM images. The ellipsometry data were fitted by a Cody Lorentz model with an effective medium approach (EMA) layer on top. The EMA layer models the surface roughness of the TiO_2_ layer. Layer thicknesses and refractive indices in the shown figures and tables refer to the thickness and dispersion obtained within the Cody Lorentz model for the amorphous part of the layer. Due to the higher number of fitting parameters, the estimated errors of the refractive index and the layer thickness are ±0.02 and ±2 nm, respectively.

The surface topology was analyzed with scanning electron microscopy (S-4800 from Hitachi, Tokyo, Japan) and atomic force microscopy (N8 TITANOS Large Sample Atomic Force Microscope from Bruker, Billerica, MA, USA). The number and the lateral size of the hillocks were determined from the SEM images (resolution: 1280 × 890 pixels, area: 11.5 × 8 µm^2^). For this purpose the greyscale SEM images were converted to binary images. In the binary image the hillocks were coded black and counted. A relative error of hillock surface coverage is estimated at 10%. It includes all errors made by the image conversion and counting. The investigated area by the AFM was 10 × 10 µm^2^ with a resolution of 1024 × 1024 pixels. From the investigation with the AFM the maximum height of the hillocks was determined. Therefore, the ten highest points of the surface were extracted. The maximum height equals the average of these ten points. The given error represents the standard deviation of this data set.

## 4. Conclusions

In this work, we identified energetic ions as the reason for surface defect formation due to the growth of polycrystalline anatase hillocks in PEALD deposited TiO_2_ films. The film growth rate and therefore the oxidation of TTiP precursor are independent of the influence on the ion flux. However, the film surface morphology and the refractive index strongly depend on the ion flux and ion energy. Defect free titania coatings can be deposited by DC biasing under plasma conditions to obtain high quality optical thin film coatings.
